# Genome-wide association studies of grain quality traits in maize

**DOI:** 10.1038/s41598-021-89276-3

**Published:** 2021-05-07

**Authors:** Yunxiao Zheng, Fan Yuan, Yaqun Huang, Yongfeng Zhao, Xiaoyan Jia, Liying Zhu, Jinjie Guo

**Affiliations:** 1 College of Agronomy, Hebei Agricultural University, Baoding, 071001 Hebei China; 2 Hebei Sub-Center of National Maize Improvement Center, Baoding, 071001 Hebei China; 3 State Key Laboratory of North China Crop Improvement and Regulation, Baoding, 071001 Hebei China

**Keywords:** Genetics, Molecular biology

## Abstract

High quality is the main goal of today’s maize breeding and the investigation of grain quality traits would help to breed high-quality varieties in maize. In this study, genome-wide association studies in a set of 248 diverse inbred lines were performed with 83,057 single nucleotide polymorphisms (SNPs), and five grain quality traits were investigated in diverse environments for two years. The results showed that maize inbred lines showed substantial natural variations of grain quality and these traits showed high broad-sense heritability. A total of 49 SNPs were found to be significantly associated with grain quality traits. Among these SNPs, four co-localized sites were commonly detected by multiple traits. The candidate genes which were searched for can be classified into 11 biological processes, 13 cellular components, and 6 molecular functions. Finally, we found 29 grain quality-related genes. These genes and the SNPs identified in the study would offer essential information for high-quality varieties breeding programs in maize.

## Introduction

Maize (*Zea mays* L.) has become one of the most important crops globally for food, feed, and fuel since it appeared and spread widely^1^. In the past few years, more and more people paid attention to the quality of maize grain due to the rapid development of animal husbandry and processing industry. However, the nutritional quality of maize grain remains poor, especially the deficiency of lysine in the maize grain, which can not meet the nutritional and health requirements of people^2^. Thus, the genetic enhancement of nutritional quality in maize grains is essential to increase the nutritional value and conduct high-quality maize breeding^2^.

The results of genetic studies have indicated that variations in nutritional quality in maize grain characterize quantitative traits. Over the past two decades, genetic dissection of nutritional quality in maize kernels by classical QTL mapping has resulted in the identification of numerous nutritional quality QTLs. Mangolin et al.^3^ detected 13 QTLs by QTL mapping of maize kernel oil content in F_2:3_ population. Liu et al.^4^ detected seven QTLs associated with protein content, six QTLs associated with starch content, and five QTLs associated with oil content using F_2:3_ population and BC_2_F_2_ population. Wang et al.^5^ detected 38 QTLs for maize grain quality traits using three RIL populations in three environments. To date, many genes related to maize proteins have been cloned, such as *opaque1* (*o1*), *floury4* (*fl4*) and *Mucronate* (*Mc*)^6–8^. Some genes such as *linoleic acid1* (*ln1*), *Oleic acid content1* (*olc1*), *fatty acid desaturation 2* (*fad2*), and *fad6* have been reported to influence oil content in maize^9–11^. A few starch content-related genes such as *Shrunken1* (*Sh1*), *Sh2* and *Brittle2* (*Bt2*) have been identified^12,13^.

Genome-wide association study (GWAS) is becoming a powerful tool to address interspecies genotype–phenotype association based on the development of next-generation sequencing technology. In maize, GWAS has made a significant progress in the past decade. For example, Li et al.^14^ used GWAS to dissect the genetic architecture of oil biosynthesis in maize kernels. Luo et al.^15^ used GWAS to detect 57 loci significantly associated with salt tolerance, and 49 candidate genes from these loci. It can be seen that GWAS is widely used in maize. However, there is still a huge problem about how to obtain phenotypic data accurately and quickly. Traditionally, phenotyping method for grain quality, such as chemical method, is not only laborious and time-consuming, but also damages the integrity of maize kernels. By contrast, Near Infrared Reflectance Spectroscopy (NIRS) is a fast, reliable, and non-destructive method. NIRS has been increasingly used in plant phenotyping measurements, such as maize kernel starch content^16^ and wheat protein content^17^. Therefore, NIRS can fully measure maize grain nutritional quality.

Although some potential grain quality genes and QTLs have been identified in maize, the genetic studies of grain quality are limited. In this study, we used NIRS to measure the main nutritional quality traits of 248 maize inbred lines and used 83,057 single nucleotide polymorphism (SNPs) markers to conduct GWAS. Our study was designed to accomplish the following objectives: (1) perform GWAS to identify SNPs responsible for moisture, protein, oil, starch and lysine contents in maize kernels, (2) compare our GWAS results with previous QTL mapping results, and (3) predict and identify candidate genes of these quality traits for future studies.

## Results

### Phenotypic variations of grain quality traits

**Figure 1:**
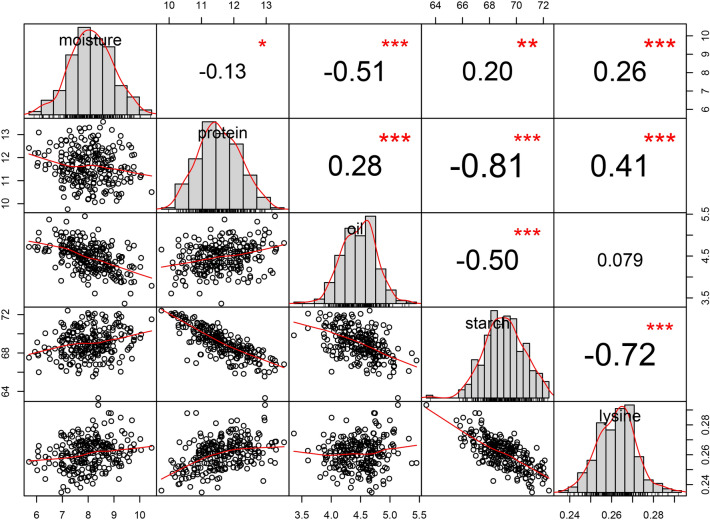
Correlation analysis of grain quality traits at BLUP. ^*^, ^**^ and ^***^ are significant correlation at *P* < 0.05, *P* < 0.01, *P* < 0.001, respectively.

The phenotypes of grain quality traits are shown in Tables 1 and 2 and Fig. 1. As displayed in Table 1, the results indicated that there were abundant phenotypic variations in the 248 inbred lines and all grain quality traits followed a normal distribution, which benefited the dissection of the genetic architecture of the grain.

**Table 1:** Statistical analysis of grain quality traits in different environments.

Trait	Environment	Range	Mean	SD	Skewness	Kurtosis	CV(%)
Moisture content (%)	2016BD	4.24–12.78	8.03	1.60	0.17	− 0.25	19.88
	2017BD	3.78–11.34	6.97	1.31	0.13	0.03	18.75
	2016SJZ	5.41–16.17	10.52	1.83	0.03	− 0.40	17.38
	2017SJZ	3.16–11.77	6.59	1.79	0.41	− 0.10	27.21
Protein content (%)	2016BD	9.22–16.71	11.96	1.28	0.38	0.11	10.70
	2017BD	8.44–14.12	11.23	1.27	0.07	− 0.62	11.31
	2016SJZ	8.85–15.75	11.75	1.29	0.34	− 0.03	11.01
	2017SJZ	8.53–14.80	11.36	1.39	0.34	− 0.41	12.23
Oil content (%)	2016BD	2.13–5.69	4.25	0.62	− 0.41	0.44	14.64
	2017BD	2.77–5.89	4.32	0.59	− 0.04	− 0.24	13.75
	2016SJZ	2.39–6.24	4.72	0.62	0.38	0.60	13.05
	2017SJZ	2.42–6.76	4.60	0.57	0.22	1.09	12.32
Starch content (%)	2016BD	59.86–75.12	69.16	2.37	− 0.52	1.01	3.42
	2017BD	58.47–74.94	69.52	2.46	− 0.49	1.32	3.54
	2016SJZ	60.15–74.84	68.83	2.65	− 0.37	− 0.03	3.86
	2017SJZ	61.24–74.84	69.05	2.70	− 0.17	− 0.33	3.91
Lysine content (%)	2016BD	0.21–0.34	0.26	0.02	0.32	0.91	7.14
	2017BD	0.23–0.33	0.27	0.02	0.52	1.14	6.34
	2016SJZ	0.17–0.33	0.24	0.02	0.52	0.63	9.58
	2017SJZ	0.22–0.32	0.27	0.02	0.34	0.54	6.58

BD and SJZ stand for Baoding and Shijiazhuang.

**Table 2:** Analysis of variance (ANOVA) for grain quality traits.

Trait	F-value				
	Environment	Genotype	Environment*Genotype	${h}^{2}$(%)	
Moisture content (%)	594.97**	4.72**	1.22*	74.19	
Protein content (%)	20.46**	5.63**	1.13	79.94	
Oil content (%)	78.03**	5.17**	1.39**	78.43	
Starch content (%)	3.39*	5.49**	1.08	80.37	
Lysine content (%)	164.79**	3.86**	1.15	70.16	

*and ** are significant correlation at *P* < 0.05 and *P* < 0.01, respectively.

Significant correlations were detected among grain quality traits, except for the correlation between moisture content and protein content (*p* = − 0.13) and the correlation between oil content and lysine content (*p* = 0.079) (Fig. 1). In addition, starch content had significant negative correlation with protein content, oil content, and lysine content, whereas the correlation among the remaining traits was positive.

Analysis of variance (ANOVA) indicated that highly significant variations for genotypes and environments were found (Table 2). However, the genotype-by-environment interaction was not significant except for the genotype-by-environment interaction of moisture content and oil content. The broad-sense heritability (${h}^{2}$, %) for grain quality traits across the four environments in the 248 inbred lines ranged from 70.16 (lysine content) to 80.37 (starch content), indicating the predominant role of genetic factors in determining these traits (Table 2). Overall, the grain quality exhibited significant genetic variations and it was suitable for association analysis.

### Genome-wide association analysis

**Figure 2:**
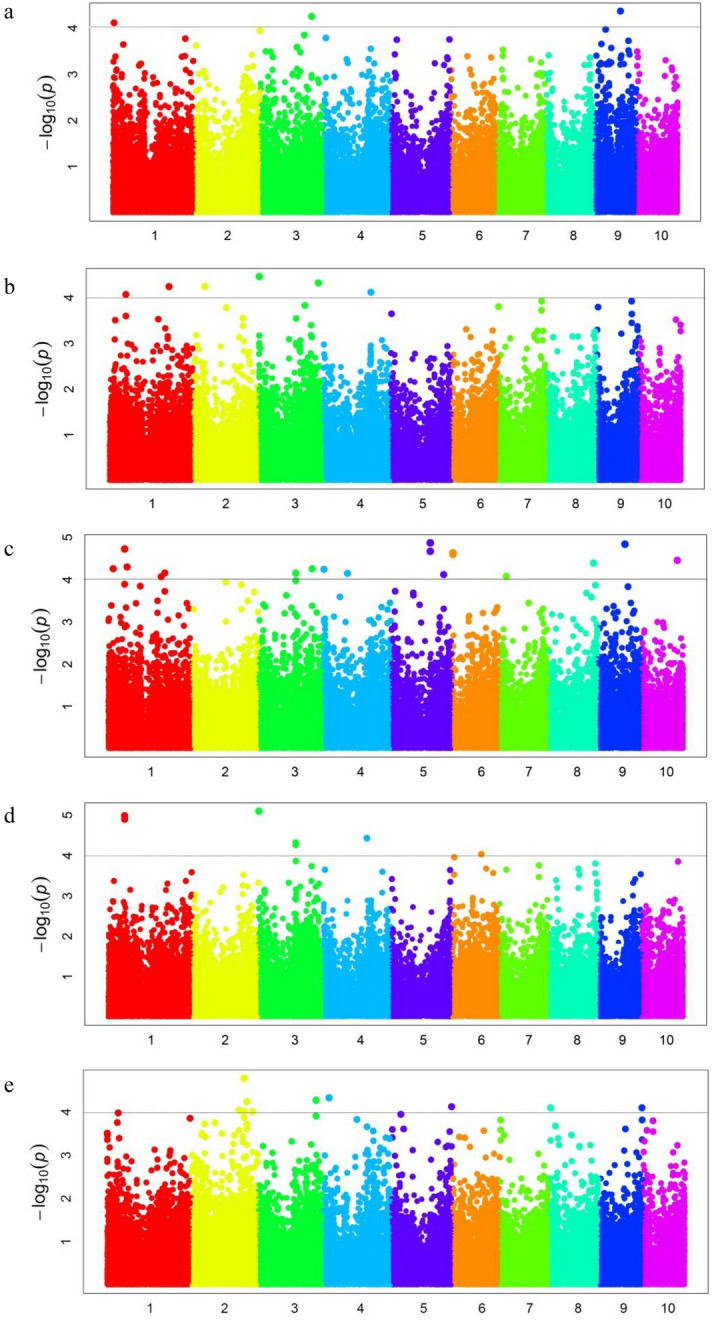
Manhattan plots of GWAS results for (**a**) moisture content, (**b**) protein content, (**c**) oil content, (**d**) starch content and (**e**) lysine content. Black solid lines correspond to the threshold (− log_10_ (*p*) = 4).

With the BLUP value of each grain quality trait, we conducted a GWAS with 83,057 genome-wide SNPs. In total, we detected 3, 7, 21, 8 and 10 SNPs to be significantly associated with moisture content, protein content, oil content, starch content and lysine content, respectively (Table 3, Fig. 2). For moisture content, three SNPs were located on chromosomes 1, 3, and 9, which individually explained 10.53%–23.16% of the phenotypic variation. For protein content, seven SNPs were located on chromosomes 1, 2, 3, and 4, which individually explained 5.45%–32.79% of the phenotypic variation. For oil content, 21 SNPs were located on chromosomes 1, 3, 4, 5, 6, 7, 8, 9, and 10, which individually explained the phenotypic variation of 9.04–31.24%. For starch content, eight SNPs were located on chromosomes 1, 3, 4, and 6, which individually explained the phenotypic variation of 3.77–23.61%. For lysine content, ten SNPs were located on chromosomes 2, 3, 4, 5, 8, and 9, which individually accounted for 5.71%–32.42% of the phenotypic variation.

**Table 3:** Analysis of correlated SNP with grain quality traits at BLUP.

Trait	SNP	Chromosome	Position	Allele	Bin	*P*-value	PVE (%)
Moisture content (%)	1_6157557	1	6,157,557	C/T	1.01	7.72E-05	10.53
	3_186486719	3	186,486,719	A/T	3.06	5.62E-05	19.09
	9_97538609	9	97,538,609	A/G	9.03	4.31E-05	23.16
Protein content (%)	1_60098312	1	60,098,312	A/G	1.04	8.54E-05	13.48
	1_215331409	1	215,331,409	C/G	1.07	5.73E-05	9.19
	2_43189825	2	43,189,825	C/T	2.04	5.71E-05	32.79
	3_1462112	3	1,462,112	A/T	3.00	3.45E-05	5.45
	3_1462147	3	1,462,147	A/G	3.00	3.45E-05	5.45
	3_213791486	3	213,791,486	C/G	3.08	4.75E-05	29.23
	4_170516460	4	170,516,460	C/G	4.06	7.63E-05	22.61
Oil content (%)	1_19252635	1	19,252,635	A/C	1.02	5.48E-05	9.08
	1_60098266	1	60,098,266	C/T	1.04	1.88E-05	9.60
	1_69088187	1	69,088,187	A/C	1.04	4.98E-05	13.13
	1_190758142	1	190,758,142	C/T	1.06	8.46E-05	20.72
	1_203725036	1	203,725,036	C/T	1.07	6.90E-05	18.61
	3_133182128	3	133,182,128	C/T	3.05	6.95E-05	22.52
	3_191700651	3	191,700,651	A/G	3.07	5.52E-05	27.91
	4_1983562	4	1,983,562	C/T	4.01	5.66E-05	29.20
	4_85966198	4	85,966,198	C/T	4.05	7.09E-05	24.09
	5_140417153	5	140,417,153	C/G	5.04	1.35E-05	14.23
	5_140417208	5	140,417,208	A/T	5.04	1.35E-05	14.23
	5_140417226	5	140,417,226	C/G	5.04	2.14E-05	13.49
	5_188466075	5	188,466,075	C/G	5.05	7.54E-05	14.36
	6_3988838	6	3,988,838	G/T	6.00	2.52E-05	9.60
	6_3988856	6	3,988,856	G/T	6.00	2.30E-05	9.56
	7_25691920	7	25,691,920	A/G	7.02	8.28E-05	17.78
	8_160425695	8	160,425,695	A/G	8.06	4.02E-05	31.24
	8_160425698	8	160,425,698	A/G	8.06	4.02E-05	31.24
	8_160425707	8	160,425,707	C/G	8.06	4.02E-05	31.24
	9_97538609	9	97,538,609	A/G	9.03	1.44E-05	17.51
	10_127895301	10	127,895,301	A/G	10.04	3.47E-05	9.04
Starch content (%)	1_60098266	1	60,098,266	C/T	1.04	1.26E-05	16.70
	1_60098312	1	60,098,312	A/G	1.04	1.02E-05	16.64
	3_1462112	3	1,462,112	A/T	3.00	7.94E-06	3.77
	3_1462147	3	1,462,147	A/G	3.00	7.94E-06	3.77
	3_133206087	3	133,206,087	A/G	3.05	4.79E-05	4.03
	3_133206096	3	133,206,096	C/G	3.05	5.43E-05	4.16
	4_155610021	4	155,610,021	G/T	4.06	3.70E-05	23.61
	6_105156642	6	105,156,642	C/T	6.04	9.22E-05	18.69
Lysine content (%)	2_169733611	2	169,733,611	A/G	2.06	9.07E-05	5.71
	2_187885582	2	187,885,582	C/T	2.07	9.82E-05	25.57
	2_187885602	2	187,885,602	A/C	2.07	1.68E-05	32.42
	2_197453621	2	197,453,621	A/T	2.07	5.84E-05	14.33
	2_218460194	2	218,460,194	C/G	2.08	9.90E-05	7.67
	3_206605349	3	206,605,349	C/T	3.08	5.39E-05	15.78
	4_20547939	4	20,547,939	C/T	4.03	4.72E-05	16.47
	5_215054319	5	215,054,319	A/T	5.08	7.60E-05	29.81
	8_4512414	8	4,512,414	C/T	8.01	8.06E-05	18.91
	9_153526598	9	153,526,598	A/G	9.07	8.07E-05	16.91

Comparing the localization results, we found eight co-localized sites and the physical position between SNPs was not more than 200 Kb (Table 4). Notably, four co-localized sites between different traits were detected. 1_60098266-60,098,312 was associated with oil content, starch content and protein content, which explained 9.60%, 16.70%, 16.64% and 13.48% of the phenotypic variation, respectively. 3_1462112-1,462,147 was associated with starch content and protein content, which explained 3.77% and 5.45% of the phenotypic variation, respectively. 3_133182128-133,206,096 was associated with oil content and starch content, which accounted for 22.52%, 4.03% and 4.16% of the phenotypic variation, respectively. 9_97538609-97,538,609 was associated with moisture content and oil content, which explained up to 23.16% and 17.51% of the phenotypic variation, respectively.

**Table 4:** Co-localized SNPs of grain quality traits in natural population.

Number	Interval	Traits	SNP	Chr	position	Allele	bin	P-value	PVE (%)	D-value (Kb)
1	1_60098266-60,098,312	oil	1_60098266	1	60,098,266	C/T	1.04	1.88E-05	9.60	0.046
		starch	1_60098266	1	60,098,266	C/T	1.04	1.26E-05	16.70	
		starch	1_60098312	1	60,098,312	A/G	1.04	1.02E-05	16.64	
		protein	1_60098312	1	60,098,312	A/G	1.04	8.54E-05	13.48	
2	2_187885582-187,885,602	lys	2_187885582	2	187,885,582	C/T	2.07	9.82E-05	25.57	0.020
		lys	2_187885602	2	187,885,602	A/C	2.07	1.68E-05	32.42	
3	3_1462112-1,462,147	protein	3_1462112	3	1,462,112	A/T	3.00	3.45E-05	5.45	0.035
		starch	3_1462112	3	1,462,112	A/T	3.00	7.94E-06	3.77	
		starch	3_1462147	3	1,462,147	A/G	3.00	7.94E-06	3.77	
		protein	3_1462147	3	1,462,147	A/G	3.00	3.45E-05	5.45	
4	3_133182128-133,206,096	oil	3_133182128	3	133,182,128	C/T	3.05	6.95E-05	22.52	23.968
		starch	3_133206087	3	133,206,087	A/G	3.05	4.79E-05	4.03	
		starch	3_133206096	3	133,206,096	C/G	3.05	5.43E-05	4.16	
5	5_140417153-140,417,226	oil	5_140417153	5	140,417,153	C/G	5.04	1.35E-05	14.23	0.073
		oil	5_140417208	5	140,417,208	A/T	5.04	1.35E-05	14.23	
		oil	5_140417226	5	140,417,226	C/G	5.04	2.14E-05	13.49	
6	6_3988838-3,988,856	oil	6_3988838	6	3,988,838	G/T	6.00	2.52E-05	9.60	0.018
		oil	6_3988856	6	3,988,856	G/T	6.00	2.30E-05	9.56	
7	8_160425695-160,425,707	oil	8_160425695	8	160,425,695	A/G	8.06	4.02E-05	31.24	0.012
		oil	8_160425698	8	160,425,698	A/G	8.06	4.02E-05	31.24	
		oil	8_160425707	8	160,425,707	C/G	8.06	4.02E-05	31.24	
8	9_97538609-97,538,609	moisture	9_97538609	9	97,538,609	A/G	9.03	4.31E-05	23.16	0.000
		oil	9_97538609	9	97,538,609	A/G	9.03	1.44E-05	17.51	

### Candidate genes associated with significant SNPs

Previous study have shown that the correlation coefficient (r^2^) between SNP markers is less than 0.1, which is considered to be no correlation^18^. Therefore, we choose r^2^ = 0.1 as the LD decay distance. Candidate genes were predicted based on LD decay (r^2^ = 0.1) in the MaizeGDB genome browser. A total of 208 candidate genes were found and detailed descriptions were summarized in Table S1.

**Figure 3:**
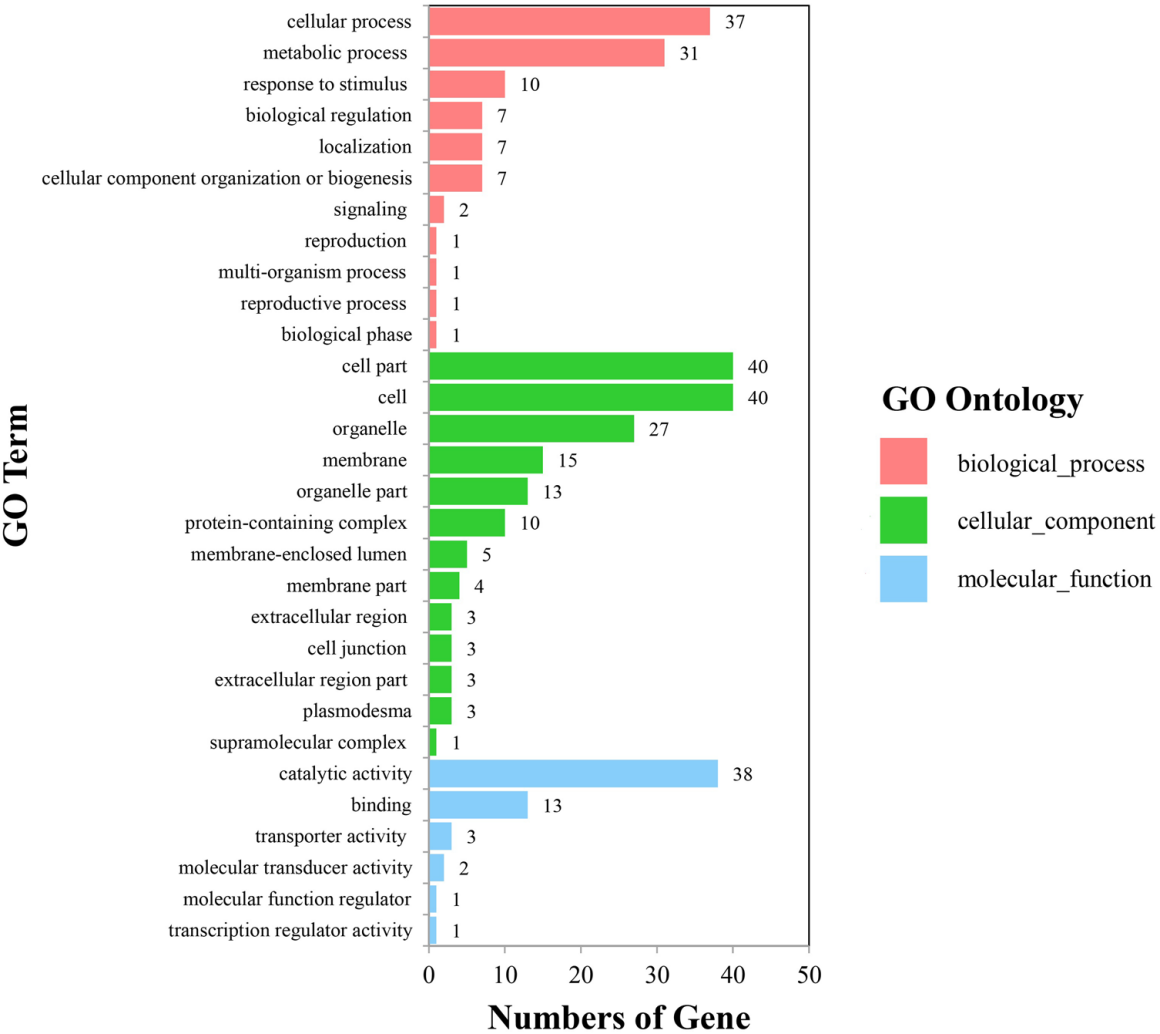
GO-second class of candidate genes.

**Figure 4:**
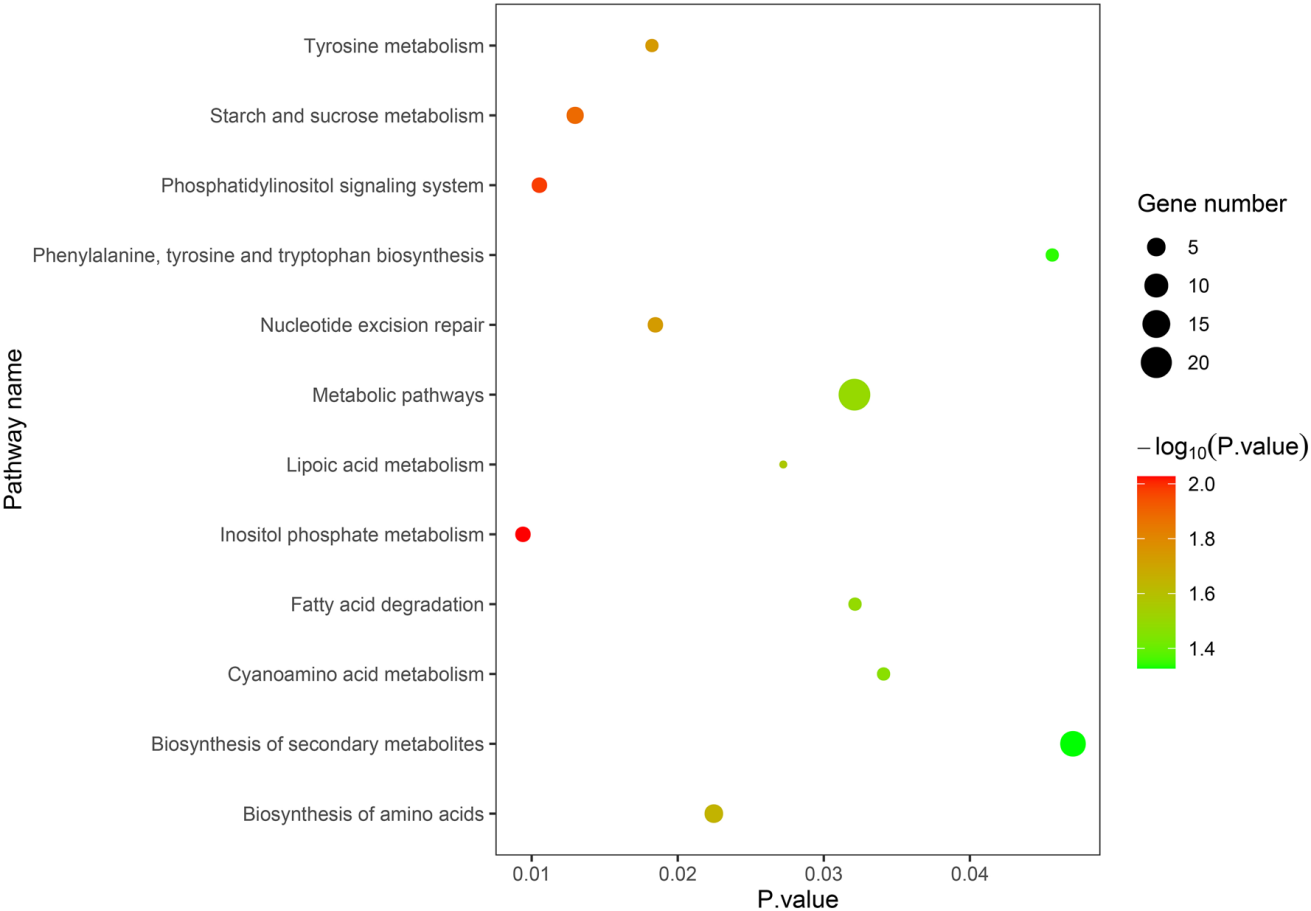
Analysis of KEGG pathway based on candidate genes. (The figure was created by R version 3.6.1 based on KEGG pathway database www.kegg.jp/kegg/kegg1.html).

**Figure 5:**
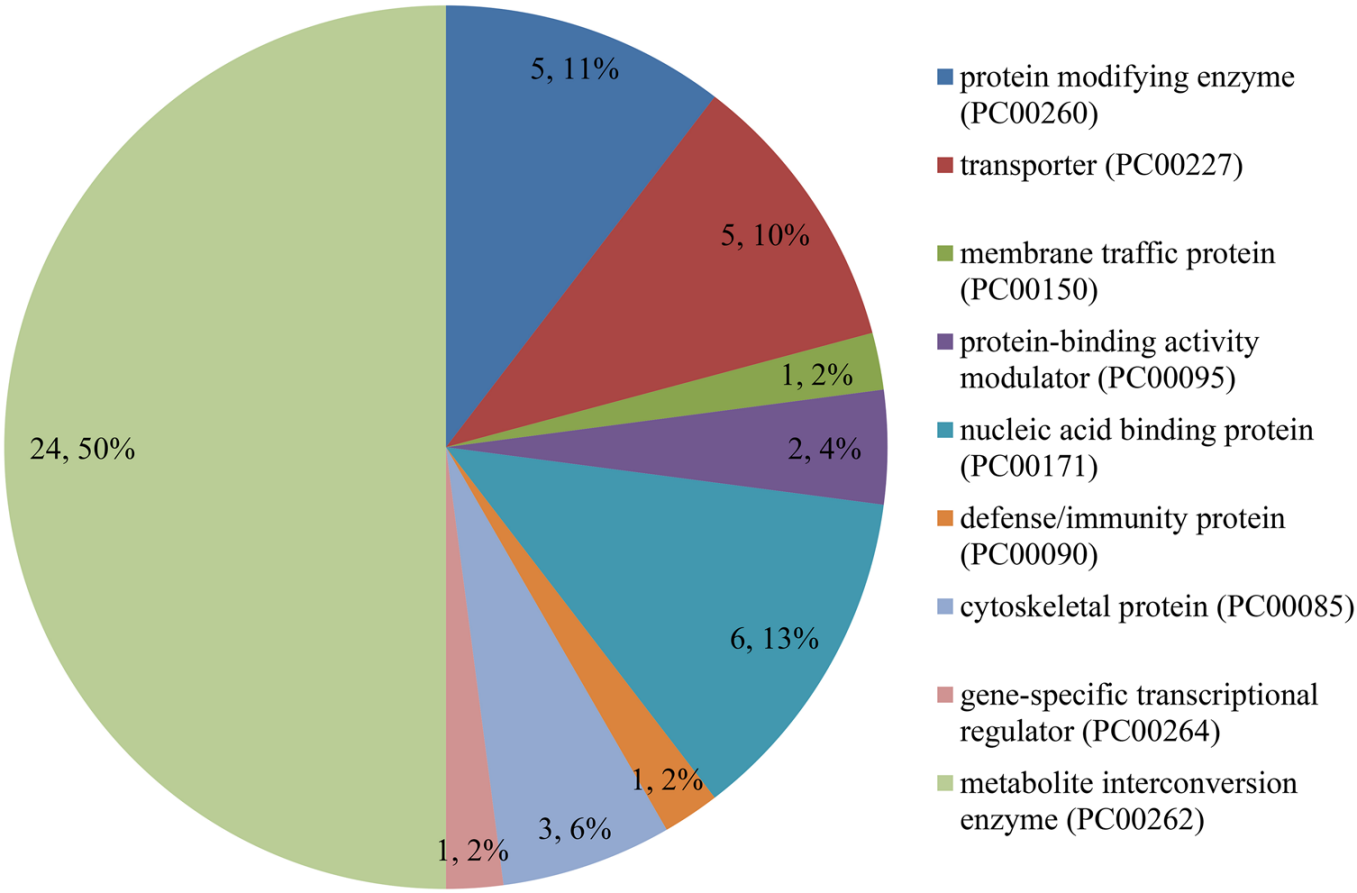
Protein classification of candidate genes.

The candidate genes can be classified into 11 biological processes, 13 cellular components, and six molecular functions. The number of candidate genes involved in the grain quality traits of moisture, protein, oil, starch and lysine contents was 77, 46, 103, 136 and 49, respectively. Among them, the candidate genes in biological processes were mainly concentrated in the cellular process and the metabolic process; the candidate genes in cellular component were mainly concentrated in organelle, cell and cell part; and the candidate genes in molecular function were mainly concentrated in catalytic activity and binding (Fig. 3). As for the KEGG analysis of the candidate genes, a total of 12 pathways were identified. These pathways included the biosynthesis of secondary metabolites, and that of amino acids, starch and sucrose metabolism, inositol phosphate metabolism, and phosphatidylinositol signaling system, which could be related to grain quality (Fig. 4). In addition, a protein classification analysis tool was used to classify candidate gene proteins, 46 of which matched the PANTHER database. A further analysis showed that these 46 proteins fell into nine categories (Fig. 5), which contained the largest number of proteins— metabolite interconversion enzyme (PC00262). Furthermore, we identified 29 candidate genes to be associated with grain quality (Table 5). Annotation information suggested that these candidate genes may control multiple traits during maize growth and development.

**Table 5:** Putative candidate gene of grain quality traits.

Trait	SNP	Candidate gene	Gene ID	RefGen_v2 Annotated Gene description
Moisture content (%)	3_186486719	*GRMZM2G069024*	100,216,811	Beta-glucosidase 11
	1_6157557	*GRMZM2G032852*	100,383,301	Putative calcium-dependent protein kinase family protein
		*GRMZM2G321041*	100,192,077	Putative RING zinc finger Domain superfamily protein
Protein content (%)	3_213791486	*GRMZM2G047129*	100,285,541	Alpha-L-fucosidase 2
	2_43189825	*GRMZM2G466833*	100,272,900	Malate dehydrogenase3
		*GRMZM2G071714*	100,279,807	Lipoyl synthase, Mitochondrial
Oil content (%)	4_1983562	*GRMZM2G033544*	103,652,693	Cyclopropane-fatty-acyl-pHospholipid synthase
	8_160425695, 8_160425698, 8_160425707	*GRMZM2G433942*	100,191,906	Palmitoyltransferase ZDHHC9
		*GRMZM2G134308*	100,384,778	Putative Beta-14-xylosyltransferase IRX10L
Starch content (%)	3_1462112, 3_1462147	*GRMZM2G175218*	100,192,000	Beta amylase4
	1_60098312, 1_60098266	*GRMZM2G082034*	100,284,904	Beta-amylase
		*GRMZM2G347708*	103,644650	Inactive beta-amylase 9
	4_155610021	*GRMZM2G000520*	103,653910	Ethylene-responsive Transcription factor ERF027
	6_105156642	*GRMZM2G404453*	103,631308	Ethylene-responsive Transcription factor ERF036
Lysine content (%)	3_206605349	*GRMZM2G050570*	100,283397	Threonine synthase
	2_218460194	*GRMZM2G129209*	100,281245	Omega-3 fatty acid Desaturase
		*GRMZM2G076307*	100,194354	Glycosyltransferases

## Discussion

### Genetic basis of grain quality traits

Maize grain quality traits are complex quantitative traits, controlled by main effect genes and lots of micro effect genes. In this study, there was a wide variety of grain quality traits in the natural population, which were normally distributed. To reduce the influence of the environment on the genotype, phenotypic BLUP values across four environments were used for association studies. Phenotypic correlations were observed among the five grain quality traits. For instance, oil content had significant positive correlation with protein content and significant negative correlation with starch content, which is consistent with previous results^4,19^. Meanwhile, starch content had significant positive correlation with protein content and lysine content, which is consistent with previous studies^20,21^. Moreover, all of the five grain quality traits had higher broad-sense heritability. Among them, the heritability for protein content, oil content, starch content and lysine content was higher than that in previous studies^21^. The above results indicated a stable genetic association among these grain quality traits of maize.

It is well known that there are hard choices between yield and quality. Previous studies showed negative correlation between quality traits and yield^22^. Therefore, how to carry out quality breeding while continuing to improve the yield of maize will be a new subject for maize breeders in the twenty-first century. At present, maize quality breeding is mainly to increase protein content and improve the composition of base acids, especially to increase the content of essential amino acids such as lysine and tryptophan^23^. In this study, a total of 83,057 SNP markers were used to scan the whole genome, combined with moisture, protein, starch, oil, lysine content and other phenotypic traits and genotypes for association analysis. The purpose of this study was to find the main genes to control the quality traits of maize, and then to introduce the genes into the parents of maize high-yield hybrids from molecular level, and then to obtain high-yield and high-quality hybrids and to provide a theoretical basis for genetic improvement of the quality traits of maize.

### Significant SNPs for grain quality traits

Nowadays many researchers at home and abroad apply linkage analysis to locating the QTL of regulating grain quality traits, but few GWAS studies are on grain quality traits. In addition, the QTL detected by linkage analysis and association analysis have consistency in position^24^. The GWAS analysis is performed with a Bonferroni correction, however this was found to be too strict for less significant trait associations. In order to better detect micro-effect polygenes and identify genetic sites, we reduced the significance threshold to -log_10_(P) = 4 for all traits^25^. In this study, a total of 49 SNPs significantly associated with grain quality were detected, and the phenotypic variation explained (PVE) value by a single SNP ranged from 3.77% (3_1462112 and 3_1462147 of starch content) to 32.79% (2_43189825 of protein content). In addition, four co-localized SNPs were detected by multiple traits and single phenotypic variation explained value over 3.77%, indicating that starch content, protein content, oil content, and moisture content are interrelated in the components of corn kernels.

The SNPs detected in this study were compared with previous studies, and some SNPs were found to be located in the localized QTL confidence interval. Among them, five SNPs were located in the QTL interval with Zhang et al.^26^, where 1_190758142 for oil content was located on chromosomes 1 (bnlg2086-umc1122 interval), 4_85966198 for oil content was located on chromosomes 4 (phi096-bnlg1755 interval), co-localized site 1_60098266-60,098,312 for protein content and starch content was located on chromosomes 1 (phi001-umc1988 interval). Two SNPs were located in the QTL interval with Wang et al.^27^, where 1_19252635 for oil content was located on chromosome 1 (umc1685-umc1044 interval), and 1_190758142 for oil content was located on chromosome 1 (umc1395-umc2237 interval). In addition, 2_169733611 for lysine content was located on chromosome 2 (bnlg1138-umc1065 interval) of study by Zhang et al.^2^. Moreover, three SNPs were located in the QTL interval with Yang et al.^28^, where 1_190758142 for oil content was located on chromosome 1 (umc1590-bnlg1556 interval), 3_213791486 for protein content were located on chromosome 3 (umc2275-umc1594 interval), 10_127895301 for oil content was located on chromosome 10 (umc1272-bnlg1839 interval).

However, some SNPs were not found in previous studies. There are several reasons for these differences. First, the population in our investigation mightnot be different enough for grain quality. Second, different estimating methods also caused the variations. Third, these SNPs were newly discovered and needed testing further. All in all, the results of this study can serve as a reference for other studies.

### Putative genes and pathways involved in grain quality

In this study, a total of 208 candidate genes were searched, of which 17 possible candidate genes for grain quality traits were predicted.

For moisture content, three candidate genes were detected. *GRMZM2G069024* encoded Beta-glucosidase 11, an important component of the cellulase system^29^. Previous studies reported that dehydration rapidly induced polymerization of AtBG1, a beta-glucosidase^30^. *GRMZM2G032852* encoded putative calcium-dependent protein kinase family protein and *GRMZM2G321041* encoded putative RING zinc finger domain superfamily protein. The two proteins emerged as key proteins in response to drought stresses in plants^31^. The three enzymes are closely related to moisture content, therefore, three candidate genes may affect moisture content by influencing enzymes activities and genes expression levels.For protein content, three candidate genes were detected. One of the candidate genes (*GRMZM2G047129*) encoded alpha-L-fucosidase 2. Alpha-L-fucosidase has been reported in only a few plants, such as *Arabidopsis*^,^^32^ and pea^33^. It was reported that alpha-L-fucosidase can hydrolyze fucose residues from glycoproteins^34^, thus this candidate gene may affect protein content. Another candidate gene (*GRMZM2G466833*) encoded malate dehydrogenase 3 (MDH), that is, one of the key enzymes to synthesize malic acid. MDH played a key role in many physiological metabolic pathways, such as C4 pathway, crassulacean acid metabolism, gluconeogenesis, tricarboxylic acid cycle and photosynthesis, linking the metabolism of sugars, proteins and lipids in the body^35^. Therefore, this candidate gene may affect protein content by influencing the metabolism of proteins. Finally, *GRMZM2G071714* encoded lipoyl synthase (LS) that analyzes the final step of lipoyl cofactor biosynthesis^36^. Protein lipoylation was denovo lipoylation pathway in plastids, and two octanoyltransferases and one LS provided protein lipoylation autonomy to plastids of *Arabidopsis*^37^. Therefore, this candidate gene may affect protein content by influencing the protein lipoylation.

For oil content, three candidate genes were identified. *GRMZM2G433942* encoded palmitoyltransferase ZDHHC9. Serine palmitoyltransferase (SPT) is the key enzyme of sphingolipids biosynthesis, and sphingolipids are essential components of plant cells^38^. As can be seen, it has a certain effect on the oil content of maize. *GRMZM2G134308* encoded putative beta-14-xylosyltransferase IRX10L. Xylose is a kind of glycosyl component widely found in plants. Plant glycosyltransferases are enzymes that are closely related to the metabolism of glycolipids, polysaccharides, glycoproteins, nucleic acids, plant secondary products, and so on^39^. Therefore, this candidate gene may affect oil content by influencing beta-14-xylosyltransferase activities and genes expression levels. *GRMZM2G033544* encoded cyclopropane-fatty-acyl-phospholipid synthase that is synonymous with cyclopropane fatty acid (CFA) synthase. CFA is an important membrane fatty acid in the stress-resistant mechanism and the presence of CFA can enhance membrane rigidity^40^. CFA synthase is a key enzyme regulating synthetic CFA. Therefore, this candidate gene may affect oil content by influencing CFA synthase activities and genes expression levels.For starch content, five candidate genes were identified. Three of the candidate genes (*GRMZM2G175218*, *GRMZM2G082034* and *GRMZM2G347708*) encoded beta-amylase, which was directly involved in the synthesis of starch and metabolic process of polysaccharides^41^. Two of the candidate genes (*GRMZM2G000520* and *GRMZM2G404453*) encoded ethylene-responsive transcription factor (ERF), a member of a transcription factor family involved in plant growth and environmental stress responses^42^. In addition, ethylene has been shown to affect starch biosynthesis by influencing enzymes activities and genes expression levels involved in starch synthesis in maize^43^.

For lysine content, three candidate genes were detected. *GRMZM2G050570* encoded threonine synthase which catalyzed the terminal reaction in the biosynthetic pathway of threonine^44^. From a nutritional point of view, lysine and threonine were essential amino acids in maize, and *GRMZM2G050570* may indirectly affect lysine content by influencing threonine synthase activities and genes expression levels.. *GRMZM2G129209* encoded omega-3 fatty acid desaturase which was a key enzyme for α-linolenic acid (ALA) biosynthesis^45^. Moreover, previous studies demonstrated that ALA was a crucial component in storing lipids in plants^46^. Therefore, the gene may regulate grain lysine content indirectly by regulating lipid synthesis. Finally, *GRMZM2G076307* encoded glycosyltransferases (GTs), which belong to a multi-member genes family. According to previous studies, GTs played a very important role in the growth and development of plants, such as regulating plant hormone levels, participating in the synthesis, modification and transportation of secondary metabolites in plants, and participating in plant defense reactions^47,48^. Therefore, the gene may regulate grain lysine content indirectly by regulating plant hormone and secondary metabolites synthesis.

All in all, these candidate genes are closely related to grain quality and future work will include functional validation of these genes and illustrate the molecular mechanisms for controlling grain quality in maize plants.

## Materials and methods

### Association mapping panel and genotyping

The association panel consisted of 248 diverse lines, including some excellent backbone inbred lines in China and some high-quality inbred lines introduced from abroad. Details on 248 of these lines could be found in previous studies^20^. The DNA of all the maize inbred lines were extracted using CTAB method^49^ and genotyped using Genotyping-By-sequencing (GBS) method^50^. The methods of SNP filtering and calculating linkage disequilibrium (LD) decay were described in previous studies^51^. A total of 83,057 SNPs were used and the LD decay was 120 kb (r^2^ = 0.1) in this study.

### Field experiments and phenotyping investigation

All 248 maize inbred lines of the association panel were planted in four environments, that is, Baoding in Hebei Province in 2016 and 2017, and Shijiazhuang in Hebei Province in 2016 and 2017. In each environment, all the maize inbred lines were planted in a single row plot using a randomized block design with two replications. Each experimental plot consisted of a row length of 3 m and 0.6 m between adjacent rows. After maturity, all corns except the head and tail of each row were harvested. Perten DA7200 Near Infrared Grain Analyzer was applied to determinate the moisture, protein, oil, starch and lysine contents of maize. Each material was repeatedly measured two times.

### Phenotype statistical analysis

The IBM SPSS 21.0 software was used to make the descriptive statistical analysis and the analysis of variance (ANOVA). The broad-sense heritability (${h}^{2}$) for each trait was estimated as ${h}^{2}={\sigma }_{g}^{2}/\left({\sigma }_{g}^{2}+{\sigma }_{gy}^{2}/r+{\sigma }_{e}^{2}/yr\right)$^52^, where ${\sigma }_{g}^{2}$, ${\sigma }_{gy}^{2}$ and ${\sigma }_{e}^{2}$ are genetic variance, genotype-by-environment interaction variance and error variance, respectively, $y$ is the number of environments, and $r$ is the number of replications. The “PerformanceAnalytics” package in the R software was used to perform correlations analysis. For each trait, BLUP value was evaluated by using the following mixed linear model in the “lme4” package of the R software^53^: Y = (1|rep%in%env) + (1|env) + (1|lines) + (1|env:lines), where Y stands for trait data, the parentheses indicate random effects, “1|” means groups, “:” means interactions, “lines” means all materials and “env” means the environment.

### Genome-wide association study of grain quality traits

The BLUP value and 83,057 SNPs were used to conduct the GWAS by using FarmCPU model^54^ implemented in the GAPIT package in the R software^55^, with both K and Q matrix taken into account.

### Prediction of candidate genes

Candidate genes were predicted based on the significant SNPs and their extension regions from 120 kb upstream to 120 kb downstream (LD decay) in the MaizeGDB (https://www.maizegdb.org/) genome browser B73 reference genome version v2. The MaizeGDB, NCBI (https://www.ncbi.nlm.nih.gov/) and Uniprot (https://www.uniprot.org/) were used to obtain annotation of candidate genes. Then these candidate genes were performed GO analysis on the GENE ONTOLOGY website (http://www.geneontology.org/). The KOBAS 3.0 website (http://kobas.cbi.pku.edu.cn/kobas3/?t=1) was used to performe Kyoto Encyclopedia of Genes and Genomes (KEGG) pathway enrichment analysis^56^.

### Statement

Experimental research and field studies complies with relevant institutional, national, and international guidelines and legislation.

## Supplementary Information

Supplementary Information
